# EHMTI-0145. Health risk behaviours in medication-overuse headache: results of a population-based study

**DOI:** 10.1186/1129-2377-15-S1-B38

**Published:** 2014-09-18

**Authors:** M Westergaard, C Glümer, E Holme Hansen, RJ Jensen

**Affiliations:** 1 Department of Neurology, Danish Headache Center, Glostrup, Denmark; 2 Glostrup Hospital, Research Center for Prevention and Health, Glostrup, Denmark; 3 Department of Pharmacy, Faculty of Health and Medical Sciences University of Copenhagen, Copenhagen, Denmark

## Introduction

Medication-overuse headache (MOH) has been called a biobehavioural disorder.

## Aim

In this cross-sectional study, we investigated associations between MOH and health-related behaviours as indicated by daily smoking, physical activity, and body mass index.

## Methods

129,150 randomly selected individuals aged >16 years were given self-administered questionnaires on general health, headache frequency, over-the-counter (OTC) analgesic use, and health behaviour. Respondents with chronic headache (>15 days/month over three months) with concurrent over-the-counter analgesic intake of >15 days/month were classified as having MOH. Associations between MOH and health behaviours were analyzed by logistic regression controlled for sociodemographic factors. All analyses were adjusted for stratified sampling and non-response.

## Results

There were 68,518 respondents. Among the 1,082 people with MOH, 34.2% were daily smokers, 48.4% were physically inactive, 57.7% were overweight/obese, and 72.0% had at least one of these three health risk factors. The group with MOH was significantly different from all other respondents in terms of prevalence of these factors (p<0.0001). MOH was significantly associated with daily smoking (odds ratio [OR] 1.5), physical inactivity (OR 1.8), and obesity (OR 1.7). Having all three health risk factors was even more strongly associated with MOH (OR 3.1 in women and OR 5.5 in men).

## Conclusion

Unhealthy lifestyle may be a modifiable risk factor for headache chronification and medication overuse. Health behaviour counseling should be part of MOH management.

No conflict of interest.

